# Supplementary material for: Visual Evidence for the Recruitment of Four Enzymes with RNase Activity to the Bacillus subtilis Replication Forks
Source: Cells. 2024 Aug 20;13(16):1381. doi: 10.3390/cells13161381 (PMC11352351; doi:10.3390/cells13161381)
Supplement: Supplementary file 1 [file cells-13-01381-s001.zip › cells-3144171-supplementary.pdf]

Supplementary Material

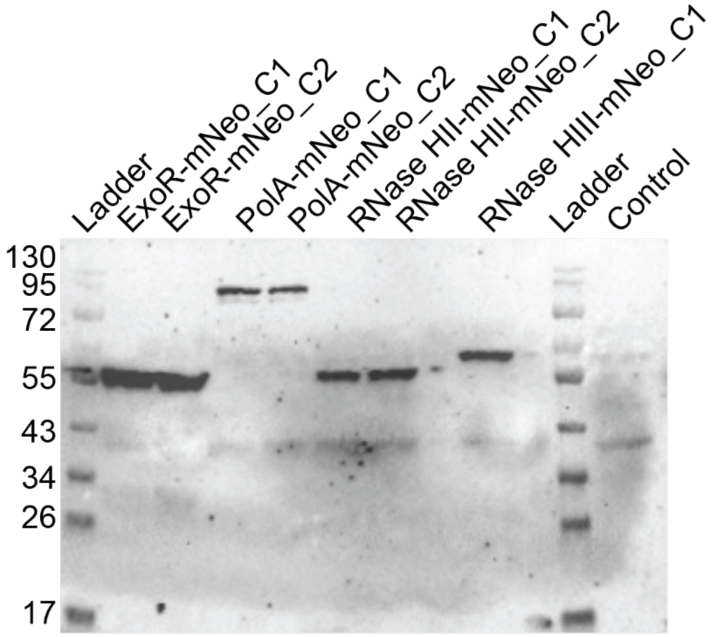

Figure S1: Western blot of potential replication proteins. Western blots showing mNeo fusion expressed from native locus. Total cell extracts from exponentially growing cultures (LB) were used. Two different clones are shown in each case (C1/C2) (except RNase HIII). The ExoR-mNeo fusion (59.7 kDa), PolA-mNeo (125.8 kDa), RNase HII-mNeo (55.1 kDa) and RNaseHIII-mNeo (60.8 kDa) contains the mNeongreen polypeptide (26.9 kDa). All strains were detected via mNeongreen-antiserum. As a control strain, the *Bacillus subtilis* BG214 was used.

A

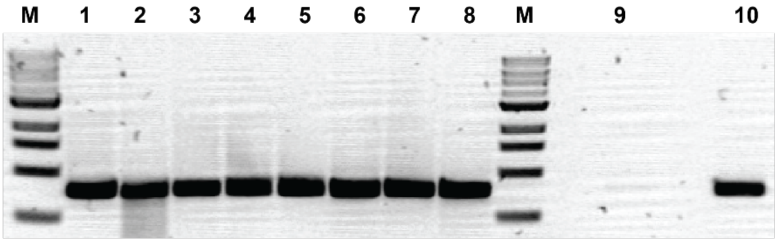

Lane	*Bacillus subtilis* BG214
1	C1_*polA*-mNeo ^{cmR} *dnaX*-CFP ^{specR} Δ*exoR*::*kan* trpC2
2	C2_*polA-mNeo* ^{cmR} *dnaX-CFP* ^{specR} Δ*exoR::kan* trpC2
3	C1_*exoR-mNeo* ^{cmR} *dnaX-CFP* ^{specR} Δ*polA::kan* trpC2
4	C2_*exoR-mNeo* ^{cmR} *dnaX-CFP* ^{specR} Δ*polA::kan* trpC2
5	C1_*rnhB*-mNeo ^{cmR} *dnaX*-CFP ^{specR} Δ*rnhC*::*kan* trpC2
6	C2_*rnhB*-mNeo ^{cmR} *dnaX*-CFP ^{specR} Δ*rnhC*::*kan* trpC2
7	C1_*rnhC*-mNeo ^{cmR} *dnaX*-CFP ^{specR} Δ*rnhB*::*kan* trpC2
8	C2_*rnhC*-mNeo ^{cmR} *dnaX*-CFP ^{specR} Δ*rnhB*::*kan* trpC2
9	*rnhB*-mNeo ^{cmR} *dnaX*-CFP ^{specR}
10	BKK40880 (Δ*exoR*::*kan* trpC2)

B

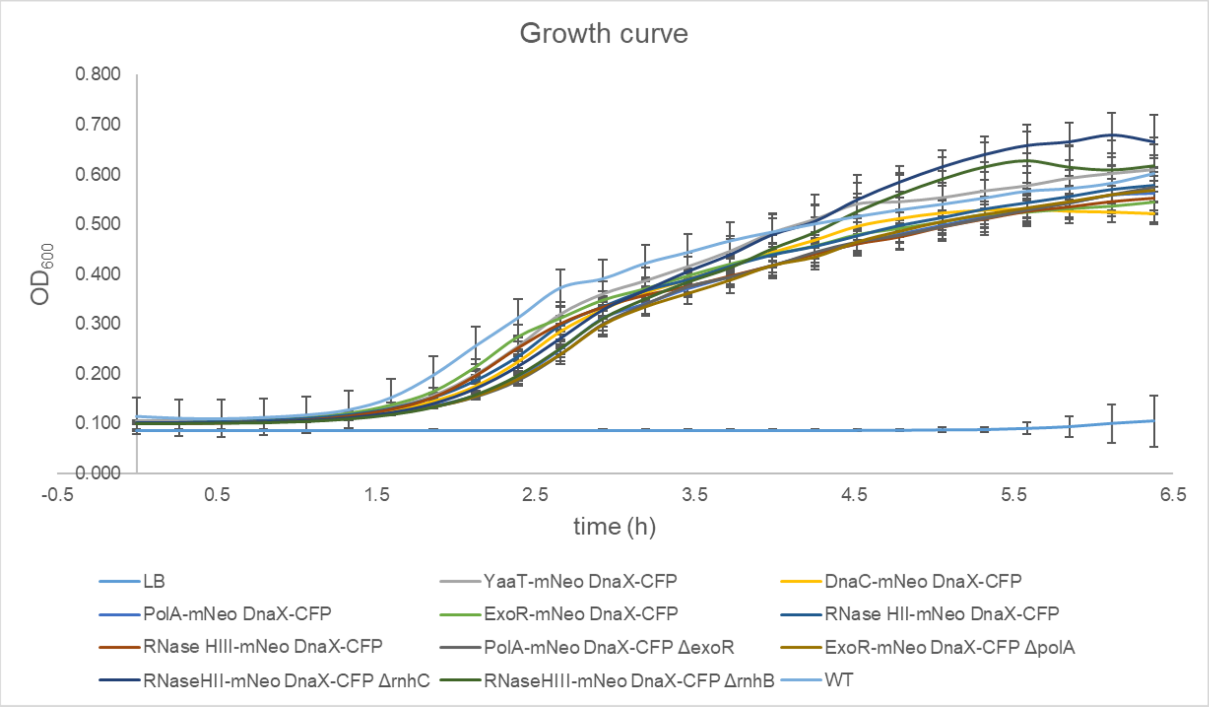

Figure S2: Functionality assay of potential replication proteins. A) Proof of gene deletion via PCR amplification of resistance cassettes. Agarose gel showing two different clones of the deletion combinations (C1/C2) besides the negative control *rnhB*-mNeo *dnaX*-CFP (lane 9) and positive control BKK40880 (lane 10). PCR was performed with specific primers binding outside the kanamycin cassette (Table S2). A size of around 750 bp was expected. **B)** Growth curves showing among the Wild type, DnaC-mNeo DnaX-CFP and LB as a negative control, mNeo fusions expressed from native locus in comparison to different deletion backgrounds. The deletions were chosen based on potential lethal combinations to proof the functionality of each fusion (PolA-mNeo DnaX-CFP with Δ*exoR*, ExoR-mNeo DnaX-CFP with Δ*polA*, RNase HII-mNeo DnaX-CFP with Δ*rnhC* and RNase HIII-mNeo DnaX-CFP with Δ*rnhB*).

Table S1: Strains or plasmids used in this study

Strain or Plasmids	Relevant features	Reference or source
Bacillus subtilis		
BG214	Wild type	gift from Juan C. Alonso
PG3977	BKK40880 (*ΔexoA*::kan trpC2)	(Koo *et al.* 2017)
PG3777	BKK28620 (*ΔrnhC*::kan trpC2)	(Koo *et al.* 2017)
PG4345	*rnhB*-mV ^{cmR}	This study
PG4346	*rnhB*-mNeo ^{cmR} *dnaX*-CFP ^{specR}	This study
PG4347	*rnhC*-mV ^{cmR}	This study
PG4348	*rnhC*-mNeo ^{cmR} *dnaX*-CFP ^{specR}	This study
PG4349	*ΔexoR*::kan trpC2 *rnhC*-mV ^{cmR}	This study
PG4350	*exoR*-mV ^{cmR}	This study
PG4351	*exoR*-mNeo ^{cmR} *dnaX*-CFP^{specR}	This study
PG4352	*ΔrnhC*::kan trpC2 *exoR*-mV ^{cmR}	This study
PG4353	*polA*-mNeo ^{cmR} *dnaX*-CFP^{specR}	This study
PG3173	*dnaX*-CFP ^{specR}	(Lindow *et al.* 2002)
PG3730	DH5α pSG1164-mVenus, expression Vektor, Amp^R, Cm^R	(Lucena *et al.* 2018)
131	pSG1164 *rnhB*-mVenus, integration Vector, Amp^R, Cm^R	This study
132	pSG1164 *rnhB*-mNeonGreen, integration Vector, Amp^R, Cm^R	This study
133	pSG1164 *rnhC*-mVenus, integration Vector, Amp^R, Cm^R	This study
134	pSG1164 *rnhC*-mNeonGreen, integration Vector, Amp^R, Cm^R	This study
135	pSG1164 *polA*-mNeonGreen, integration Vector, Amp^R, Cm^R	This study

136	pSG1164 *exoR*-mNeonGreen, integration Vector, Amp^R, Cm^R	This study
137	pSG1164 *exoR*-mVenus, integration Vector, Amp^R, Cm^R	This study

Table S2: List of Oligonucleotides

**Primer**	**Sequence 5'→ 3'**
rnhB-mNeo fw	GATTCCTAGGATGGGTACCGAATTCTTGTCGCCAGCGCAGTCATCCTTCC
rnhB-mNeo rev	CTCCCAGGCCAGATAGGCCGGGCCCTCTGAAAGATTGAACAGGAGCGAAA
rnhB_fw_full length	GTGAATACATTAACCGTAAA
rnhC-mNeo fw	GATTCCTAGGATGGGTACCGAATTCGAAACCTGATTAAAACCATTCCGTA
rnhC-mNeo rev	CTCCCAGGCCAGATAGGCCGGGCCCTGAACGTTTTTTATCAGCAAGGCGC
rnhC_fw_full length	CACCGACAATCGTGAAGTGC
exoR-mNeo fw	GATTCCTAGGATGGGTACCGAATTCCGGACATTACGGTGGTAACAGGGGA
exoR-mNeo rev	CTCCCAGGCCAGATAGGCCGGGCCCAACGATCTCTCTAGCGTTCAGCTTT
exoR-fw_full length	ATGAATAATAATAAACTATTGCTGGTTGAC
PolA-mNeo fw	GATTCCTAGGATGGGTACCGAATTCTTGGCATTGTTTACGGGATCAGCGA
PolA-mNeo rev	CTCCCAGGCCAGATAGGCCGGGCCCTTTCGCATCGTACCAAGATGGGCCT

PolA_fw_full length	CGAAAAAAATTAGTGCTTGT
DnaC-mNeo fw	GATTCCTAGGATGGGTACCGAATTCTTTACATCGATGATACACCGGGTAT
DnaC-mNeo rev	CTCCCAGGCCAGATAGGCCGGGCCCTGCGCCGGGCGGAACGCCTGCGTCA
DnaC_fw_full length	ATGACAGACCTTCTGAATGA
mNeonGreen rev	TTACTTGTACAGCTCGTCCA
KanR-fw	ATGGCTAAAATGAGAATATC
KanR-rev	CTAAAACAATTCATCCAGTA
